# From problematic screen use to stress: psychological quality and academic burnout among 10- and 13-year-olds children

**DOI:** 10.3389/fpsyg.2026.1819969

**Published:** 2026-09-01

**Authors:** Yan Xianghui, Li Keyu, Yong Huiwen, Zhang Xianglan, Cui Yifan

**Affiliations:** 1 Department of Education and Psychology, Southwest Minzu University, Chengdu, China; 2 Sichuan Minzu Educational Development Research Center, Chengdu, China

**Keywords:** academic burnout, adaptability, masking effect, problematic screen use, psychological quality

## Abstract

**Background:**

In the digital age, excessive screen-related behavior among children and adolescents has been associated with adverse psychological and academic outcomes; however, the indirect associations linking problematic screen use, psychological quality, and academic burnout remain under-explored.

**Aim:**

To examine the associations among problematic screen use, psychological quality, and academic burnout among students aged 10–13 and to test whether psychological quality and its dimensions were statistically involved in the association between problematic screen use and academic burnout.

**Method:**

A total of 476 upper-grade primary school students aged 10 and 13 were assessed using the Problematic Media Use Measure Short Form, Psychological Quality Scale, and Academic Burnout Scale. Data were analyzed with SPSS 27.0 and mediation models with Bootstrap methods.

**Results:**

Problematic Screen Use was positively associated with academic burnout (*r* = 0.48, *p* < 0.01) and negatively associated with overall psychological quality (*r* = −0.38, *p* < 0.01) and its dimensions. Psychological quality exhibited a statistical masking pattern rather than a protective indirect association, driven primarily by the adaptability dimension. Higher Problematic Screen Use scores were associated with lower adaptability, whereas adaptability was positively associated with burnout in the adjusted model; together, these coefficients produced a negative indirect association that partially offset the positive direct association. A sequential indirect-effect model identified an ordered statistical pattern involving cognitive characteristics, personality traits, and adaptability. Because the data were cross-sectional, these findings do not establish temporal ordering or causal processes.

**Conclusion:**

Within this sample, the statistical role of psychological quality appeared to be context-dependent, with adaptability contributing to an unexpected masking pattern. The findings provide preliminary evidence regarding the associations among Problematic Screen Use, Psychological Quality, and Academic Burnout in upper-grade primary school students. They should be interpreted as cross-sectional, sample-specific associations rather than evidence of developmental mechanisms or causal effects.

## 1 Introduction

In the digital era, the pervasive use of screen-based devices has become a defining feature of modern life, particularly among children and adolescents. Recent studies highlight that excessive screen exposure, termed Problematic Screen Use is associated with a range of negative outcomes, including impaired cognitive development, increased risk of mental health issues, and adverse academic performance (Straker and Howie, 2016; Przybylski and Weinstein, 2019). Specifically, upper-grade primary school students aged 10 and 13 are at a critical developmental stage, marked by significant physiological and psychological changes. This period is characterized by heightened autonomy and complex motivations for screen use in adolescent, often driven by entertainment and social interaction (Kwon et al., 2024). Concurrently, these students face escalating academic demands, making them particularly vulnerable to academic burnout – a condition defined by emotional exhaustion, detachment from learning, and a diminished sense of achievement (Vinter et al., 2020).

Recent research underscores the importance of psychological quality as a potential protective correlate of screen-related behavior and academic stress. Psychological quality, encompassing cognitive flexibility, emotional regulation, and adaptive coping strategies, has been associated with lower levels of burnout (Baumeister et al., 2007; Xu et al., 2025). However, the relationships among problematic screen use, psychological quality, and academic burnout are complex and multifaceted. Emerging evidence suggests that the association between problematic screen use and academic outcomes may statistically involve psychological resources (Kjellenberg et al., 2025). For example, cognitive overload accompanying excessive screen use may be associated with reduced mental resources, lower academic engagement, and greater burnout symptoms (Uncapher et al., 2017). Prolonged screen exposure may also co-occur with lower psychological quality and poorer academic functioning (Loh and Kanai, 2016). These theoretical possibilities do not, however, establish directionality in cross-sectional data.

Despite growing interest in this area, existing studies have predominantly focused on screen time duration as a singular metric, often neglecting the multidimensional nature of psychological quality (Crawford et al., 2010). This oversight limits our understanding of how different facets of psychological quality – such as cognitive characteristics, personality traits, and adaptability – may differentially mediate the relationship between screen exposure and academic burnout. Furthermore, research specifically targeting upper-grade primary school students remains limited, highlighting the need for more targeted investigations.

To address these gaps, the present study examines the indirect statistical associations among problematic screen use, psychological quality, and academic burnout in students aged 10–13. By adopting a multidimensional framework, this study seeks to clarify how cognitive characteristics, personality traits, and adaptability are related to the observed association between problematic screen use and academic burnout. The models are intended to characterize patterns of association rather than to establish causal or developmental processes.

## 2 Literature review

Problematic Screen Use in children and adolescents refers to the use of any screen-based device, with increasing duration and younger onset ages (Khan, 2008; Li et al., 2025). Premature or excessive screen use is linked to physiological issues such as obesity and psychological problems like anxiety and depression, with long-term impacts among children and adolescents (Twenge and Campbell, 2018). Research highlights that problematic screen use, characterized by dependency or loss of control, can displace time and cognitive resources needed for learning, rest, and social interaction (Feng et al., 2025). This can lead to cognitive overload and disrupted sleep patterns, further impairing daytime functioning (Loh and Kanai, 2016).

Academic Burnout is a chronic stress response resulting from prolonged academic pressure, characterized by emotional exhaustion, detachment from learning, and reduced self-efficacy (Wang P. et al., 2023). It is significantly predicted by academic stress (Cheng and Lin, 2023). In upper-grade primary school students, burnout often manifests as internalized skepticism about learning and emotional detachment, rather than overt resistance. This can directly affect academic performance and lead to problematic behaviors such as absenteeism (Gao, 2023). The transition to upper-grade primary school is particularly challenging as students face increased academic demands, making them more susceptible to burnout.

Psychological Quality, comprising cognitive characteristics, personality traits, and adaptability, is a key resource associated with coping under stress. However, in the context of digital stress, its statistical role may be context-dependent. Cognitive and personality resources may be associated with better management of problematic screen use and stronger resistance to distraction, whereas prolonged screen use may co-occur with lower levels of these resources and adaptability (Cerniglia and Cimino, 2025). Accordingly, dimensions of psychological quality may be statistically associated with both problematic screen use and academic burnout. Nevertheless, the direction and temporal ordering of these relationships cannot be determined from existing cross-sectional evidence.

Existing research has identified negative associations between screen exposure and academic/psychological outcomes, with some exploration of mediating psychological factors (Feng et al., 2025). However, limitations include a focus on screen duration rather than problematic screen use, lack of detailed multidimensional analyses of psychological quality, and insufficient studies on upper-grade primary school students. To plug these gaps, this study aims to examine the mediating role of psychological quality in the relationship between family screen exposure and academic burnout among upper-grade primary school students. By adopting a multidimensional theoretical framework, this study seeks to provide a more nuanced understanding of the complex interplay among these variables.

Conservation of Resources theory provides the central framework for integrating problematic screen use, psychological quality, and academic burnout. The theory proposes that individuals attempt to acquire, preserve, and protect valued resources. In the academic context, cognitive control, stable personality-related strengths, adaptive coping capacities, time, and emotional energy may function as personal resources. Problematic screen use may compete with academic activities for these resources through repeated attentional switching, displacement of study or sleep time, and demands on self-regulation. Academic burnout may be more likely when available resources are insufficient to meet persistent academic demands.

Adaptability, in most prior research operationalizes as proactive, active adjustment to academic challenges, which consistently predicts reduced stress and burnout (Xie et al., 2019; Chen et al., 2023a). However, emerging family digital exposure research proposes a distinct subtype: passive compliance adaptation, referring to children’s forced behavioral adjustment to parents’ excessive screen use norms without autonomous psychological acceptance. This passive adaptation consumes self-control resources and triggers ego depletion, generating a counter-intuitive positive link between overall adaptability and academic burnout. Few previous studies have distinguished two adaptability subtypes within a single general adaptability scale, which may explain the inconsistent cross-sectional findings (Granziera et al., 2024).

Psychological quality is therefore conceptualized as a multidimensional personal resource rather than as an isolated trait. Cognitive characteristics may support the organization and monitoring of learning, personality traits may support persistence and self-discipline, and adaptability may help students respond flexibly to academic and environmental demands. From this perspective, problematic screen use is expected to be negatively associated with psychological quality, whereas psychological quality is expected to be negatively associated with academic burnout. Psychological quality may consequently account for part of the statistical association between problematic screen-media use and academic burnout.

Accordingly, we proposed that: (H1) problematic screen use would be positively associated with academic burnout; (H2) problematic screen use would be negatively associated with psychological quality; and (H3) psychological quality and its dimensions would show indirect statistical associations between problematic screen use and academic burnout.

## 3 Aim of the study

This study aims to investigate whether psychological quality is statistically involved in the association between problematic screen use and academic burnout among upper-grade primary school students aged 10–13. Specifically, it examines indirect associations involving cognitive characteristics, personality traits, and adaptability. These regression-based indirect-effect models are used to describe theoretically specified patterns of covariance and should not be interpreted as evidence of causal mechanisms or temporal developmental pathways.

## 4 Research design

### 4.1 Participants

This study employed a cluster sampling procedure, selecting four classes from each of the fifth and sixth grades across three ordinary primary schools. A total of 500 questionnaires were distributed, and after incomplete and anomalous responses were excluded, 476 valid questionnaires were retained, yielding a valid response rate of 95.4%. The sample consisted of 209 males (43.9%) and 267 females (56.1%), with ages ranging from 10 to 13 years (M = 11.12, SD = 0.68). An *a priori* power analysis using G*Power 3.1 (effect size f^2^ = 0.15, α = 0.05, and power 1-β = 0.95) indicated a minimum required sample size of 138 for the planned regression-based analyses. The final sample of 476 exceeded this numerical requirement. However, the power analysis addresses statistical sensitivity rather than representativeness, and recruitment from only three schools limits the external validity of the findings. All participants provided informed consent, and the questionnaires were administered anonymously.

### 4.2 Measures

#### 4.2.1 Problematic screen use

The Problematic Media Use Measure Short Form (PMUM-SF), developed by Domoff et al. (2017), was adopted to measure children’s problematic screen/media use behavior. PMUM-SF specifically captures dysfunctional, addictive, and disruptive patterns of media engagement rather than usage time. Consistent with the study’s core objective, we uniformly adopt the term “problematic screen use” throughout the manuscript to align with the construct measured by this scale (Li et al., 2024). This scale was adapted for upper-grade primary school students with necessary revisions in Chinese. Comprising nine items, it uses a 5-point Likert scale (1 = Strongly Disagree, 5 = Strongly Agree), with higher scores indicating a greater tendency toward problematic screen use. In this study, the scale’s Cronbach’s α coefficient was 0.72.

#### 4.2.2 Psychological Quality Scale

The Psychological Quality Scale for Middle School Students (Revised Edition), adapted by Hu et al. (2017), was employed to measure psychological quality. Although originally developed for middle school students, we adapted this scale for upper-grade primary school students by simplifying the wording of four items (e.g., changing “I can plan my study time effectively” to “I can arrange my study time well”) to better match the cognitive level of 10- to 13-year-olds, while retaining the original three-dimensional structure. This scale includes 24 items across three dimensions: cognitive characteristics, personality traits, and adaptability. It uses a 5-point Likert scale (1 = Strongly Disagree, 5 = Strongly Agree), with higher scores indicating higher levels of psychological quality. In this study, the scale’s Cronbach’s α coefficient was 0.84.

#### 4.2.3 Academic burnout scale

Based on the Adolescent Learning Burnout Scale developed by Wu et al. (2010), an academic burnout questionnaire suitable for upper-grade primary school students was revised. The original scale was designed for secondary school students; we therefore simplified the wording of six items (e.g., changing “I feel that I cannot adapt to the learning pace in middle school” to “I feel that I cannot keep up with the learning pace”) and confirmed the revisions with two senior primary school teachers and three psychology researchers to ensure age-appropriateness and content validity. The questionnaire includes 16 items across three dimensions: emotional exhaustion, detachment from learning, and low sense of achievement. It uses a 5-point Likert scale, with higher scores indicating more severe academic burnout. In this study, the scale’s Cronbach’s α coefficient was 0.61. This relatively low reliability may be attributable to the high internal heterogeneity of the subdimensions and the restricted variance due to the narrow age range of the sample; results involving this scale should therefore be interpreted with caution.

#### 4.2.4 Confirmatory factor analyses and reliability of the measurement instruments

Confirmatory factor analyses were conducted using diagonally weighted least squares estimation because the questionnaire items were measured on five-point ordered response scales. Model fit was evaluated using the χ^2^/df ratio, comparative fit index (CFI), Tucker–Lewis index (TLI), Root Mean Square Error of Approximation (RMSEA), and Standardized Root Mean square Residual (SRMR).

The one-factor model of the nine-item Problematic Media Use Measure–Short Form showed acceptable global fit, χ^2^(27) = 90.71, χ^2^/df = 3.36, CFI = 0.947, TLI = 0.930, RMSEA = 0.070, and SRMR = 0.074. Standardized factor loadings ranged from 0.050 to 0.830. Six items had significant loadings between 0.524 and 0.830, whereas three items had weak and non-significant loadings. The global fit was therefore acceptable, but the local item-level results indicate that the construct validity of the complete nine-item score should be interpreted cautiously in this sample.

The correlated three-factor model of Psychological Quality, comprising cognitive characteristics, personality traits, and adaptability, also showed good fit, χ^2^(296) = 490.15, χ^2^/df = 1.66, CFI = 0.984, TLI = 0.982, RMSEA = 0.037, and SRMR = 0.060. Standardized loadings ranged from 0.288 to 0.723 and were statistically significant (*p* < 0.001). The administered instrument contained 26 items: seven measuring cognitive characteristics, eight measuring personality traits, and eleven measuring adaptability.

The correlated three-factor model of Academic Burnout, comprising emotional exhaustion, detachment from learning, and reduced sense of achievement, showed good fit, χ^2^(101) = 167.76, χ^2^/df = 1.66, CFI = 0.981, TLI = 0.977, RMSEA = 0.037, and SRMR = 0.056. Standardized loadings ranged from 0.371 to 0.775, and all freely estimated loadings were statistically significant (*p* < 0.001).

### 4.3 Data processing and analysis

Data analysis was conducted using SPSS 27.0 and the PROCESS 4.1 macro. First, data cleaning and preparation were performed. Outliers were identified and handled using box plots and *Z*-scores (| *Z*| > 3.29), and missing values were imputed using the series mean method. Total scores for problematic screen use, psychological quality, and academic burnout, as well as mean scores for each dimension, were calculated. Next, the Harman single-factor test was used to assess common method bias. Descriptive statistics and Pearson correlation analyses were then conducted. Regression-based indirect-effect analyses were performed using Model 4 of the PROCESS macro developed by Hayes (2017). Bootstrap sampling with 5,000 replications was used to estimate the indirect associations involving (1) overall psychological quality and (2) its three dimensions – cognitive characteristics, personality traits, and adaptability – while controlling for participant gender, grade, and age. The sequential model was treated as exploratory. Because all variables were measured at one time point, the estimated direct and indirect coefficients represent statistical associations and do not demonstrate temporal precedence or causality. All significance levels were set at *p* < 0.05.

### 4.4 Common method bias assessment

Given that all measures in this study were collected via self-report questionnaires, the potential for common method bias was considered. To address this, we employed well-validated scales with strong reliability and emphasized the anonymity of the survey during data collection. Additionally, we assessed the extent of common method bias using Harman’s single-factor test. This method involves conducting an exploratory factor analysis (EFA) on all items without rotation to determine if a single factor dominates the variance.

The results of the EFA indicated that the first unrotated factor accounted for 24.07% of the variance, which is below the commonly accepted threshold of 40% (Podsakoff et al., 2003). This suggests that common method bias is not a significant concern in this study and is unlikely to substantially influence the findings.

### 4.5 Ethical considerations

The study was approved by the department of ethics committee (Approval No. 2025-026). Before completing the questionnaire, each participating student – and their parent or legal guardian – provided written informed consent using an age-appropriate, short-form consent sheet. Students returned the finished questionnaires to school the following day. The research team then anonymized all data, conducted group-level analyses, shared aggregate findings with the school, and prepared a manuscript for public dissemination.

## 5 Results

### 5.1 Descriptive statistics and correlation analysis

Descriptive statistics and the Pearson correlation matrix for the core variables are presented in Table 1. The mean score for problematic screen use among upper-grade primary school students was 25.95 (SD = 6.19), while the mean score for academic burnout was 43.11 (SD = 8.17). The overall mean score for psychological quality was 96.10 (SD = 16.29). Correlation analysis revealed a significant positive relationship between problematic screen use and academic burnout (*r* = 0.48, *p* < 0.01). problematic screen use was also significantly negatively correlated with overall psychological quality (*r* = −0.38, *p* < 0.01) and its dimensions, including cognitive characteristics, personality traits, and adaptability. However, overall psychological quality was not significantly correlated with academic burnout. When examined by dimension, cognitive characteristics (*r* = −0.14, *p* < 0.01) and personality traits (*r* = −0.13, *p* < 0.01) were significantly negatively correlated with academic burnout, while adaptability was not significantly correlated with academic burnout. These findings suggest that problematic screen use is associated with academic burnout, and that cognitive and personality dimensions of psychological quality are related to academic burnout Among 10- and 13-year-old children.

**TABLE 1:** Descriptive statistics and Pearson correlation matrix for core variables (*N* = 476).

Variable	M(SD)	1	2	3	4	5
1 Screen exposure	25.95 (6.19)	1	1	1	1	1
2 Academic burnout	43.11 (8.17)	0.48**				
3 Psychological quality total	96.10 (16.29)	**−**0.38**	**−**0.07			
4 Cognitive characteristics	27.06 (5.45)	**−**0.35**	**−**0.14**	0.84**		
5 Personality traits	30.26 (6.29)	**−**0.36**	**−**0.13**	0.88**	0.73**	
6 Adaptability	38.78 (6.89)	**−**0.23**	0.09	0.82**	0.61**	0.61**

**p < 0.01.

### 5.2 Mediation effect of psychological quality

To examine whether psychological quality was statistically involved in the association between problematic screen use and academic burnout, a bootstrap indirect-effect analysis (PROCESS Model 4) was conducted while controlling for gender and grade. problematic screen use was specified as X, psychological quality as M, and academic burnout as Y. The results (Table 2) showed that problematic screen use was negatively associated with psychological quality (path a: B = −0.92, *p* < 0.001), and psychological quality was positively associated with academic burnout after adjustment for problematic screen use (path b: B = 0.06, *p* = 0.004). The adjusted direct association between problematic screen use and academic burnout was significant (path c’: B = 0.69, *p* < 0.001). The estimated indirect association was −0.06, with a 95% bootstrap confidence interval of [−0.11, −0.01]. Because the total and direct associations were positive whereas the indirect association was negative, this coefficient pattern was statistically consistent with a masking effect (MacKinnon et al., 2000). This terminology describes the observed regression pattern and does not imply a causal process.

**TABLE 2:** Mediation effect of psychological quality (*N* = 476).

Path	Effect	*SE*	*t*	*p*	95% CI
a (Screen Exposure → Psychological Quality)	**−**0.92	0.11	**−**8.09	<0.001	[**−**1.14, **−**0.69]
b (Psychological Quality → Academic Burnout)	0.06	0.02	2.88	0.004	[0.02, 0.10]
c’ (Screen Exposure → Academic Burnout)	0.69	0.06	12.21	<0.001	[0.58, 0.80]
Total Effect (c)	0.63	0.05	11.87	<0.001	[0.53, 0.74]
Indirect Effect (a × b)	**−**0.06	0.02			[**−**0.11, **−**0.01]

SE = Standard Error; CI = Confidence Interval.

### 5.3 Multidimensional mediation effects of psychological quality

To further examine the statistical roles of the components of psychological quality, three separate indirect-effect models were estimated using cognitive characteristics, personality traits, and adaptability as candidate mediators. The results (Table 3) showed that only the indirect association involving adaptability was significant, with an estimate of −0.06 and a 95% bootstrap confidence interval of [−0.11, −0.03]. Problematic screen use was negatively associated with adaptability (path a: B = −0.25, *p* < 0.001), whereas adaptability was positively associated with academic burnout after adjustment for problematic screen use (path b: B = 0.24, *p* < 0.001). The indirect associations involving cognitive characteristics and personality traits were not significant. Thus, the masking pattern in the total-score model was statistically concentrated in the adaptability dimension. This finding should be understood as a model-dependent association rather than evidence that problematic screen use reduces adaptability or that adaptability causally increases burnout.

**TABLE 3:** Multidimensional mediation effects of psychological quality (*N* = 476).

Mediator (*M*)	Path a (X → *M*)	Path b (*M*→ Y)	Direct effect (c’)	Total effect (c)	Indirect effect (a × b)	Bootstrap 95% CI	Significant-Yes or No
Cognitive characteristics	**−**0.30	0.05	**−**0.65***	0.63***	**−**0.01	[**−**0.06, 0.03]	No
Personality traits	**−**0.36	0.07	0.66***	0.63***	**−**0.02	[**−**0.07, 0.02]	No
**Adaptability**	**−0.25**	0.24***	0.70***	0.63***	**−0.06**	**[−0.11, −0.03]**	**Yes**

Path a, b, c’, c, and indirect effect values are unstandardized coefficients. Bootstrap CI = bias-corrected confidence interval.

*** p < 0.001. Bold values indicate significant mediation effects.

Table 3 indicates that only the model involving adaptability produced a significant indirect association. The negative indirect coefficient partially offset the positive adjusted association between problematic screen use and academic burnout; it should not be interpreted as evidence of a protective or harmful causal pathway.

Notably, while correlation analysis revealed significant negative associations between cognitive characteristics, personality traits, and academic burnout (Table 1), these dimensions did not emerge as significant mediators in the separate mediation models. This apparent inconsistency can be understood from two complementary perspectives.

First, the non-significant overall correlation between total psychological quality and academic burnout likely reflects the canceling out of dimension-specific effects. While cognitive characteristics and personality traits exert protective effects (evidenced by negative correlations), these may be offset or suppressed by the opposing influence of adaptability, which shows a risk-enhancing pattern (positive correlation). When aggregated into a total score, these contradictory effects attenuate the overall association, resulting in a non-significant relationship between total psychological quality and academic burnout.

Second, after statistically controlling for problematic screen use, the unique associations of cognitive characteristics and personality traits with academic burnout (path b) became non-significant. This pattern may indicate substantial shared variance among problematic screen use, psychological resources, and burnout. However, the present cross-sectional models cannot establish that screen exposure is a common cause of these variables. The results instead demonstrate that bivariate correlations and adjusted regression coefficients capture different aspects of their covariance structure.

### 5.4 Prediction of academic burnout by psychological quality: hierarchical and multiple regression analyses

To clarify the independent contributions and relative importance of screen exposure and the dimensions of psychological quality to academic burnout, hierarchical regression analysis was conducted, controlling for gender, grade, academic performance, and caregivers. The results of the hierarchical regression analysis (Table 4) are as follows:

**TABLE 4:** Hierarchical regression analysis (*N* = 476).

Predictor	Model 1	Model 2	Model 3
First layer: control variables			
Gender	**−**0.06	**−**0.05	**−**0.04
Grade	**−**0.06	**−**0.08	**−**0.08*
Academic performance	**−**0.15**	**−**0.09	**−**0.12*
Caregiver	**−**0.07	0.04	0.01
Second layer: core predictor			
Screen exposure		0.48***	0.49***
Third layer: dimensions of psychological quality			
Cognitive characteristics			**−**0.10
Personality traits			**−**0.04
**Adaptability**			0.32***
*R* ^2^	0.04	0.25	0.31
Δ*R*^2^		0.21***	0.06***
Adjusted *R*^2^	0.03	0.24	0.30

*p < 0.05, **p < 0.01, ***p < 0.001. Regression coefficients in bold indicate statistical significance.

In the first step, the model explained a limited amount of variance in academic burnout (R^2^ = 0.04) after including demographic and control variables. In the second step, the addition of screen exposure significantly increased the model’s explanatory power (ΔR^2^ = 0.21, *p* < 0.001), confirming problematic screen use as a core risk factor for academic burnout. In the third step, the inclusion of the three dimensions of psychological quality (cognitive characteristics, personality traits, and adaptability) further enhanced the model’s explanatory power, albeit to a lesser extent (ΔR^2^ = 0.06, *p* < 0.001). In the final model, screen exposure (β = 0.49, *p* < 0.001) and adaptability (β = 0.32, *p* < 0.001) emerged as the most significant positive predictors of academic burnout, while cognitive characteristics and personality traits did not show significant predictive effects. This finding underscores the unique role of adaptability in the model and its counter-intuitive direction of effect.

### 5.5 Moderation and chain mediation analysis of psychological quality

#### 5.5.1 Moderation analysis

Using Process Model 1, we examined whether the total score of psychological quality moderated the relationship between problematic screen use and academic burnout. The results indicated that the interaction term between problematic screen use and total psychological quality did not significantly predict academic burnout (B = 0.01, *p* = 0.06, 95% CI [−0.001, 0.014]). Thus, no significant moderating effect of total psychological quality was found, suggesting that the impact of problematic screen use on academic burnout does not vary significantly across different levels of psychological quality.

#### 5.5.2 Chain mediation analysis

Chain mediation was assessed using Process Model 6 to explore the sequential effects within psychological quality. The specific indirect effects and their significance from the Bootstrap analysis (5000 samples) are summarized in Table 5.

**TABLE 5:** Specific indirect effects in chain mediation model (*N* = 476).

Path	Mediation path	Effects	Boot SE	Bootstrap 95% CI	Significant-Yes or No
Ind1	Screen Exposure → Cognitive Characteristics → Academic Burnout	0.05	0.03	[**−**0.00, 0.12]	No
Ind2	Screen Exposure → Personality Traits → Academic Burnout	0.01	0.01	[**−**0.01, 0.05]	No
Ind3	Screen Exposure → Adaptability → Academic Burnout	0.01	0.02	[**−**0.02, 0.06]	No
Ind4	Screen Exposure → Cognitive Characteristics → Personality Traits → Academic Burnout	0.02	0.02	[**−**0.02, 0.07]	No
**Ind5**	**Screen Exposure → Cognitive Characteristics → Adaptability → Academic Burnout**	**−0.05**	**0.02**	[**−0.10, −0.02]**	Yes
**Ind6**	**Screen Exposure → Personality Traits → Adaptability → Academic Burnout**	**−0.02**	**0.01**	[**−0.05, −0.00]**	Yes
**Ind7**	**Screen Exposure → Cognitive Characteristics → Personality Traits → Adaptability → Academic Burnout**	**−0.03**	**0.02**	[**−0.08, −0.02]**	Yes

Effect values are unstandardized coefficients; CI = bias-corrected Bootstrap confidence interval. Bold values indicate significant mediation paths.

**FIGURE 1:**
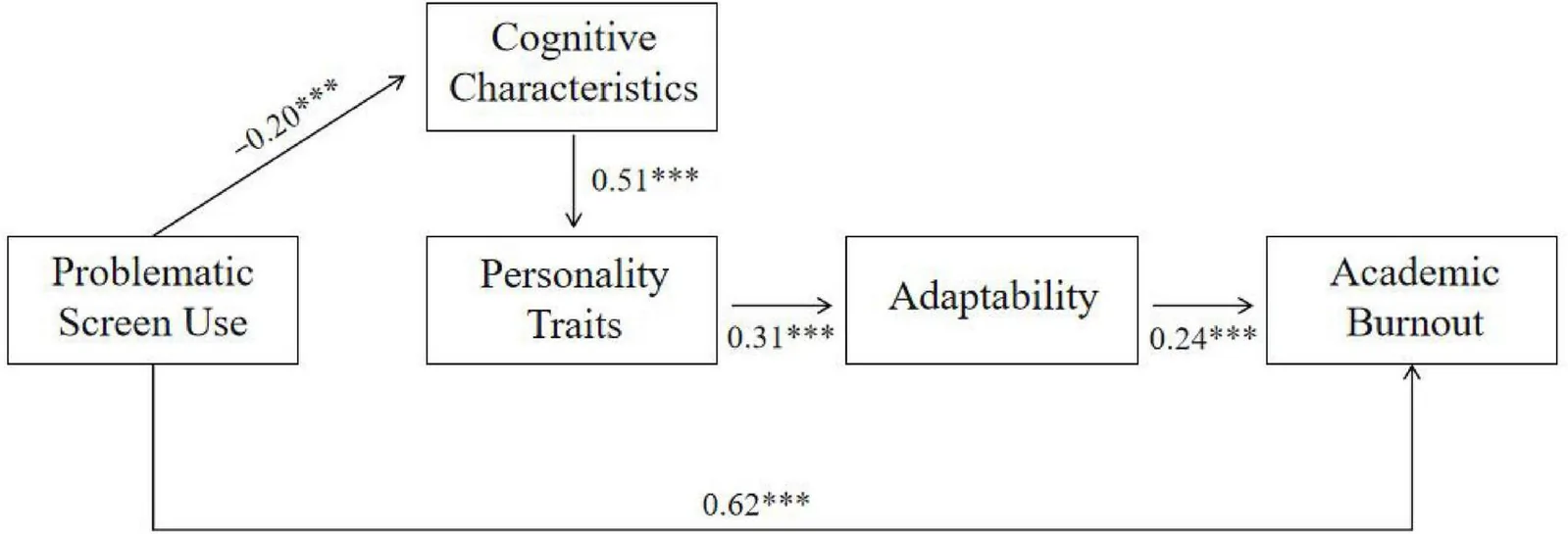
Standardized path coefficients for the chain mediation model. ***p < 0.001.

To visually summarize the chain mediation findings, Figure 1 presents the standardized path coefficients for the sequential model. As shown in Figure 1, the indirect paths from problematic screen use to academic burnout via cognitive characteristics → personality traits → adaptability were all significant, with the final segment (adaptability → academic burnout) showing a positive coefficient (β = 0.24, *p* < 0.001), consistent with the masking pattern identified in the single-mediator analyses.

All coefficients are standardized (*p* < 0.001. Direct paths from cognitive characteristics and personality traits to academic burnout were non-significant and are omitted for clarity. The indirect paths via adaptability all showed significant negative indirect effects, consistent with the masking pattern identified in the single-mediator analyses.

The chain mediation model revealed a sequential pathway within psychological quality: problematic screen use negatively impacted cognitive characteristics, which in turn affected personality traits and adaptability. Ultimately, only adaptability significantly and positively predicted academic burnout. Bootstrap analysis showed that three paths ending with adaptability (Ind5, Ind6, Ind7) had significant negative indirect effects. For instance, the path from screen exposure to cognitive characteristics to adaptability to academic burnout had an effect of −0.05 (95% CI [−0.10, −0.02]). This finding clarifies the mechanism of the masking effect: problematic screen use reduces cognitive characteristics and personality traits, ultimately diminishing adaptability. Since lower adaptability is associated with lower levels of academic burnout, this sequence statistically offsets part of the direct harmful effect of screen exposure.

## 6 Discussion

This study examined the associations among problematic screen use, psychological quality, and academic burnout in upper-grade primary school students. Problematic screen use was positively associated with academic burnout, consistent with previous studies identifying excessive digital media use as a potential risk marker (Valkenburg and Peter, 2013; Twenge and Campbell, 2018). The analyses also identified an unexpected statistical masking pattern involving psychological quality, particularly adaptability. Because the data were cross-sectional, the discussion below offers possible theoretical interpretations of these associations rather than verified causal explanations.

### 6.1 Digital transformation of the masking effect between problematic screen use and psychological quality

The finding that problematic screen use significantly and positively predicts academic burnout aligns with studies linking excessive digital media use to decreased academic engagement and emotional exhaustion among adolescents (Twenge and Campbell, 2018; Rana and Kaur, 2020). However, the observed masking effect of overall psychological quality suggests a fundamental shift in its function under chronic, high-intensity digital media stress.

From the perspective of the Conservation of Resources (COR) theory, individuals are motivated to acquire, retain, and protect valuable resources such as time, energy, and positive self-concept (Hobfoll, 1989). Problematic screen use creates a continuous threat to these resources through constant information flow, multitasking, and social comparison (Halbesleben et al., 2014). High psychological quality individuals, rather than having inexhaustible resources, may be more adept at resource allocation strategies. When faced with inescapable academic demands and screen temptations, they may strategically divert cognitive and emotional resources from leisure, sleep, or deep thinking to maintain immediate academic performance and behavioral compliance. This “robbing Peter to pay Paul” strategy may sustain functional adaptation in the short term but can lead to long-term resource depletion (Hobfoll, 2001). Statistically, this excessive effort for adaptation manifests as a positive association between psychological quality and academic burnout, weakening the visible association between problematic screen use and burnout, thus creating a masking effect. This mechanism is similar to the masking effect of psychological depletion between job demands and burnout (Crawford et al., 2010).

Self-regulation theory offers another explanatory pathway. Psychological quality, particularly cognitive characteristics (planning, monitoring) and personality traits (self-discipline, perseverance), is closely related to high self-regulation ability (Baumeister et al., 2007). However, self-regulation is a limited resource that can be exhausted through frequent use, known as ego-depletion (Baumeister et al., 1998). The screen use environment in China, filled with immediate rewards (e.g., short videos, game feedback) and distracting stimuli, demands constant inhibition of attentional impulses and redirection to academic tasks, a high-intensity self-regulation process (Uncapher et al., 2017). High psychological quality students may experience more severe self-regulation resource depletion in this process. After expending significant self-regulation resources to resist screen/media temptations and adapt to the learning environment, they have fewer resources left to cope with academic setbacks and manage negative emotions, making them more prone to burnout. Thus, high psychological quality may indicate a rapidly depleting self-regulation system rather than a well-stocked one, with its positive correlation with burnout reflecting this internal depletion state.

Developmental contextualism emphasizes that development is the result of continuous interaction between individual characteristics and multiple environments (Lerner, 2018). The digital media environment is a novel and highly pervasive developmental context. Our findings suggest that in the specific context of current digital ecology intertwined with high-intensity academic pressure, the expression and functional consequences of traditionally adaptive psychological quality are reshaped. In this context, good adaptation may be forced to bind with maladaptive strategies such as excessive self-control and emotional suppression. Therefore, high scores in psychological quality may reflect an individual’s successful internalization and adaptation to the demands of this specific context, which itself is a risk factor for burnout.

### 6.2 Duality of adaptability as a core variable

The masking effect is primarily driven by the adaptability dimension. Understanding the paradox that higher adaptability is associated with higher burnout requires a deep understanding of the multidimensionality and context sensitivity of adaptability.

In the measurement domain, widely used adaptability scales typically assess overall functional levels in social and academic domains but often fail to distinguish the nature of strategies used to achieve this functionality. Extensive research differentiates coping strategies into active, problem-focused strategies and passive, emotion-focused strategies (Connor-Smith et al., 2000). In contexts where problematic screen use reality and academic pressure cannot be changed, high adaptability scores may be associated with more frequent use of secondary control strategies (cognitive restructuring, acceptance) or surrender strategies (avoidance, self-blame). While the former has some adaptiveness, long-term reliance may lead to weakened control; the latter is explicitly related to psychological distress (Skinner and Zimmer-Gembeck, 2007). The adaptability in our study may capture this complex adaptive pattern mixed with acceptance and submission. A study on Chinese adolescent psychological resilience also found that certain resilience traits may be associated with higher depressive symptoms under extreme stress, indicating the existence of a dark side of resilience (Oshio et al., 2018). This results indicated the duality of Adaptability – autonomous adaptation and passive context-bound compliance – in Chinese context.

This theoretical differentiation between active autonomous adaptation and passive context-bound compliance aligns with recent research distinguishing heterogeneous adaptive strategies (Granziera et al., 2024; Chen et al., 2023b). Adaptability is a context-bound construct whose adaptive or detrimental psychological outcomes hinge heavily on surrounding environmental conditions. Focusing on the combined stressful context of chronic problematic screen use and rigorous academic pressure among 10- and 13-year-olds in this study, we argue that high scores on a general adaptability scale do not inherently represent healthy proactive adjustment. To illustrate, adolescents may force themselves to abandon personal leisure and extend studying hours to cope with fierce academic rivalry, or constantly adjust their online behaviors and social media identities to accommodate family and peer screen/media-related expectations. While such behavioral compliance allows children to functionally fit into their stressful surroundings, this repeated forced adjustment triggers ego depletion and compromises long-term psychological well-being, which we define as passive compliance adaptation within a harmful contextual environment. Correspondingly, our findings urge scholars to move beyond generalized, unidimensional measurements of adaptability. Instead, a contextualized, strategy-specific evaluation system is required to distinguish autonomous active adaptation from costly passive compliance adaptation, avoiding overgeneralized interpretations based merely on a global adaptability total score.

In Addition, cultural psychology believed that the expression and evaluation of adaptability are deeply rooted in cultural norms. Collectivist culture emphasizes harmony, obedience, and academic achievement, which may shape a unique adaptive prototype: adjusting oneself to meet family and societal expectations (Chen and French, 2008). In the Chinese educational context, well-adapted students are often expected to show high academic compliance, diligence, and emotional restraint. This culturally scripted adaptation differs from the more autonomy-focused, self-expressive adaptation emphasized in individualistic cultures (Markus and Kitayama, 1991). Therefore, high adaptability individuals in our study may be those who have more deeply internalized the good student role, are more sensitive to academic pressure, and are more inclined to cope with conflicts through self-suppression. This explains why their adaptability scores are positively correlated with intrinsic exhaustion (closely related to academic burnout). Leung et al.’s study also supports that high adherence to traditional values (e.g., filial piety) may increase psychological pressure in adolescents under specific contexts (Leung et al., 2010).

### 6.3 Chain erosion and masking pathways of psychological quality dimensions

The chain mediation model reveals the dynamic process of digital pressure eroding students’ psychological health: “problematic screen use → cognitive characteristics → personality traits → adaptability → academic burnout.” This chain model can be further explained from the perspectives of cognitive neuroscience and personality development.

The first stage (damaging cognitive characteristics) has a solid cognitive science basis. Problematic screen use, especially entertainment-oriented, multi-platform, high-stimulation use, is closely related to sustained attentional distraction, increased working memory load, and decreased deep processing ability (Uncapher et al., 2017). Neuroimaging studies suggest that frequent media multitasking is associated with changes in the structure and function of the anterior cingulate cortex and prefrontal cortex, which are core areas responsible for cognitive control and attentional allocation (Loh and Kanai, 2016). Therefore, problematic screen use directly affects students’ subjective cognition in learning, which may lead to academic burnout.

The second stage (undermining personality traits) reflects the spillover effect of cognitive resource depletion on the motivational and volitional systems. Traits such as perseverance and self-discipline in personality traits are not static personality traits; their manifestation highly depends on available cognitive resources (Duckworth et al., 2019). When cognitive resources are continuously depleted by combating screen/media distractions, students’ ability to maintain long-term learning goals, resist immediate temptations, and persist in setbacks (i.e., grit) decreases. This aligns with the strength model of self-control, where all forms of self-control share the same limited resource pool (Baumeister et al., 2007). Cognitive fatigue caused by screen exposure directly depletes the psychological energy supporting personality traits.

The third stage (weakening adaptability and creating masking) is the most theoretically novel part of our model. The weakening of adaptability here should be precisely understood as the impoverishment of the adaptive strategy repertoire or a shift toward more passive, emotion-focused strategies. When both cognitive and volitional resources are in short supply, the range of effective coping strategies available to individuals drastically narrows. They may shift from active problem-solving (planning screen use time, seeking help) to more passive, emotion-focused coping (avoidance, fantasizing, self-blame) (Compas et al., 2014). In our model, the positive prediction of adaptability on burnout may mark the dominance of such passive coping strategies. The chain pathway ultimately reduces this adaptability associated with high burnout (actually reducing harmful passive coping tendencies), creating a negative indirect effect that masks the direct harm of screen exposure. This is not true protection but reflects the terminal state of a decay chain: when an individual’s psychological system decays comprehensively from cognition to motivation to adaptive strategies, their response to external pressure (problematic screen use) may become numb or desensitized, presenting lower burnout in self-reports, which is actually a sign of psychological dysfunction, not a healthy symbol.

### 6.4 Cultural heterogeneity in problematic screen use: reward-oriented screen time in Chinese households

A wealth of prior research has documented that adaptive functioning typically serves as a protective factor, correlating with less academic pressure and fewer burnout symptoms among young students. Yet our subgroup analyses show contrasting connections between adaptability and academic burnout depending on the degree of household screen exposure, a pattern that does not align with the uniform protective relationship observed in most overseas research. It is worth noting that our study only adopted a unified general adaptability scale and lacked dedicated measurement tools to separately capture active proactive adjustment and passive compliance adaptation. Interpretations centered on compliance-driven adjustment and ego depletion can only be indirectly supported by subgroup correlation differences, rather than direct latent variable evidence. Beyond the psychological costs of mandated context-based adjustment, cross-cultural variations in how families regulate problematic screen use provide an additional contextual perspective to interpret these mixed statistical outcomes.

Scholars from Western contexts mostly treat household digital engagement as a homogeneous risk factor, holding the view that increased screen use inevitably raises psychological distress and learning difficulties (Nagata et al., 2025). Nevertheless, screen use activities among Chinese upper-grade primary students carry two distinct practical functions, molded by the locally pervasive exam-focused parenting logic (Wang C. Y. et al., 2023). Large-scale national surveys targeting Chinese minors further verify that linking screen access to academic performance stands as a mainstream parental media control tactic. Caregivers commonly restrict children’s right to use screens (e.g., smartphones, streaming platforms or televisions), until all school assignments are completed, or offer extra digital entertainment time as positive feedback when youngsters reach expected academic benchmarks (Cui et al., 2026).

Under such standardized family rules, appropriate screen engagement acts as a controllable, target-guided motivating factor instead of an overwhelming psychological burden. For children who possess autonomous, positive adaptive capabilities, their robust ability to adjust to surroundings allows them to steadily follow household academic requirements, maintain steady study schedules, and obtain planned digital rewards on a regular basis, which in turn eases their feelings of academic exhaustion. This culturally unique reward mechanism helps explain why adaptability displays disparate correlations with burnout in groups split by household screen use intensity, and casts doubt on the one-sided linear risk assumption generalized from Western adolescent samples.

### 6.5 Practical implications

The findings may have tentative implications for educational practice, but these implications should not be interpreted as causal, evidence-based intervention effects established by the present cross-sectional study. Rather than regarding higher psychological quality and general adaptability as uniformly beneficial for adolescent academic development, educators and parents are advised to evaluate students’ adaptive outcomes within their specific family digital contexts and to distinguish the underlying behavioral and psychological strategies of adaptation (Livingstone, 2018). Targeted practical support includes fostering adolescents’ digital literacy, improving sustained attention management, establishing healthy daily screen-use routines, cultivating flexible self-regulation and psychological recovery strategies, and enriching accessible social support resources to buffer screen-related psychological strain (Livingstone et al., 2018). Furthermore, families and schools should reduce over-reliance on academic achievement as the sole evaluation criterion for student development and provide adequate opportunities for children’s autonomous exploration and holistic psychological growth. Importantly, these practice-oriented suggestions are theoretically inferred rather than empirically validated; their practical effectiveness requires rigorous verification through dedicated intervention research in future studies.

### 6.6 Limitations and future directions

This study has several notable limitations that should be acknowledged, which also suggest targeted directions for future investigation. First, the cross-sectional design precludes rigorous temporal sequencing and causal inferences. Although the PROCESS models revealed conditional direct and indirect associations among variables, we cannot confirm the directional hypothesis that problematic screen use precedes changes in students’ psychological quality and academic burnout. Reverse or reciprocal relationships are highly plausible: for instance, students suffering from severe academic burnout may rely more on screen use for emotional relief, and pre-existing psychological vulnerabilities may simultaneously predict maladaptive screen behaviors and burnout symptoms. To address this limitation, future studies can adopt longitudinal cross-lagged designs, daily diary protocols, or controlled experimental paradigms to clarify the temporal ordering and potential reciprocal relationships among problematic screen use adaptive patterns, psychological quality, and academic burnout, so as to establish more robust causal inferences. Second, all study variables were measured via student self-report questionnaires, which are susceptible to recall bias and social desirability effects, potentially compromising the objectivity of the results.

Accordingly, future research is strongly recommended to adopt multi-source data collection approaches, incorporating parent and teacher ratings, objective device-based screen-use records, and behavioral observations to replace single self-report data and mitigate reporting bias. Third, the adaptability construct was assessed only through a unidimensional general scale, failing to differentiate proactive autonomous adaptation and passive compliance adaptation. As a result, our theoretical interpretation of passive adaptation and ego depletion underlying the counter-intuitive adaptability–burnout association is merely supported by indirect subgroup correlational evidence, rather than direct latent variable measurement. Future work should therefore develop and validate a refined two-dimensional adaptability scale that distinguishes active adaptive tendencies and passive compliance strategies, while integrating ego depletion as a mediating variable to systematically verify the psychological mechanism proposed in the present study. Fourth, the generalizability of the findings is constrained by the sampling strategy. The final sample of 476 fifth- and sixth-grade students was recruited from only three ordinary primary schools. Despite sufficient statistical power for regression-based analyses, the limited school coverage restricted the reliable estimation of school-level nested effects and may have reduced the precision of standard errors, making the results unrepresentative of broader student populations, diverse geographical regions, and different educational and cultural contexts. In the future, researchers should recruit larger, multisite, and socio-culturally diverse samples and apply multilevel or cluster-adjusted statistical analyses to adequately address nested data structures and improve external validity. Finally, cross-cultural replications and preregistered intervention trials are warranted to further test the robustness and translational value of the current findings across diverse educational and cultural contexts.

## 7 Conclusion

Based on a sample of 476 10- and 13-year-olds children in three ordinary primary schools, the present study explored the associations between problematic screen use, psychological quality, and academic burnout. The results confirmed a positive linkage between problematic screen use and academic burnout and further revealed an unexpected statistical masking effect exerted by psychological quality. After full model adjustment, such masking patterns were predominantly manifested in the dimension of student adaptability. The sequential analysis further identified a systematic covariation pattern among cognitive characteristics, personality traits, adaptability, and academic burnout. Importantly, this study only detected correlational covariance rather than solid causal pathways or progressive psychological erosion mechanisms. Given the cross-sectional design and constrained sampling scope, the current findings remain preliminary and context-specific, requiring further validation through larger and more diverse samples.

In summary, this study highlights the inherent complexity of the interplay between problematic screen use, multidimensional psychological quality, and academic burnout among upper-grade primary school students. The counterintuitive results particularly underscore the necessity of adopting context-sensitive and strategy-differentiated evaluations of student adaptability, rather than generalized, one-dimensional judgments. Nevertheless, no universal conclusions regarding child and adolescent populations can be drawn from the present evidence. Accordingly, the educational implications proposed in this study should be treated as tentative and exploratory until robust replications are achieved through longitudinal, multisite, and multilevel methodological designs. Future intervention practices should rely on rigorous empirical verification instead of direct inferences derived from the cross-sectional correlational results observed in this study.

## Data Availability

The datasets generated and/or analyzed during the current study are available from the corresponding author on reasonable request. Requests to access these datasets should be directed to doc.yan@vip.163.com.
